# Reply to Comments: “Molecular Functions of Thyroid Hormone Signaling in Regulation of Cancer Progression and Anti-Apoptosis” *Int. J. Mol. Sci.*, 2019, *20*, 4986

**DOI:** 10.3390/ijms21103554

**Published:** 2020-05-19

**Authors:** Yu-Chin Liu, Chau-Ting Yeh, Kwang-Huei Lin

**Affiliations:** 1 Department of Biochemistry, College of Medicine, Chang-Gung University, Taoyuan 333, Taiwan; k1506820@gmail.com; 2 Department of Biomedical Sciences, College of Medicine, Chang-Gung University, Taoyuan 333, Taiwan; 3 Liver Research Center, Chang Gung Memorial Hospital, Taoyuan 333, Taiwan; chauting@adm.cgmh.org.tw; 4 Research Center for Chinese Herbal Medicine, College of Human Ecology, Chang Gung University of Science and Technology, Taoyuan 333, Taiwan

Dear Editor,

In Dr. Magdalena Szaryńska’s recent letter, she expressed one confusing aspect she met in Figure 1 [1]. We had already corrected and published on [2], circulating thyroid hormones (THs) interact with thyroid hormone receptors to promote downstream signaling pathways and activate transcription factors. Thyroid hormone receptors (TR) including TRα and TRβ contain several domains; specifically, these are the amino-terminal A/B that may function as a gene enhancer, the DNA-binding domain (DBD), the hinge region containing the nuclear localization signal and the carboxy-terminal ligand-binding domain (LBD) that binds T3 through AF-2, which is a surface-exposed hydrophobic included residue from H3 and H5 and is completed by T3-dependent packing of C-terminal H12 against the LBD which affects target genes transcription [3,4]. (Figure 1). The four major TR isoforms, TRα1, TRα2, TRβ1, and TRβ2, are produced by *c-erbA*α and *c-erbAβ* genes. Their human homologs are designated THRA and THRB. The *c-erbA*α gene located on chromosome 17 encodes two different TRα isoforms. One is functional TH-binding TRα1 and the other is a dominant-negative splice variant, TRα2, lacking TH binding activity [5]. T3 interacts with thyroid hormone receptors via C-terminal activation function-2 (AF-2) in the ligand-binding domain (LBD), however, only TRα2 has a distinct C-terminal extension and is absent the activation function-2 (AF-2) region, which suggested that TRα2 does not bind T3 [6]. TRα2 is unique in consideration of its lack of binding to THs while interacting with DNA, and its precise function is unclear at present. We have corrected to show that the TRα2 didn’t bind T3 and have marked the presence or absence of the AF-2 domain in Figure 1 as follows:

## Figures and Tables

**Figure 1:**
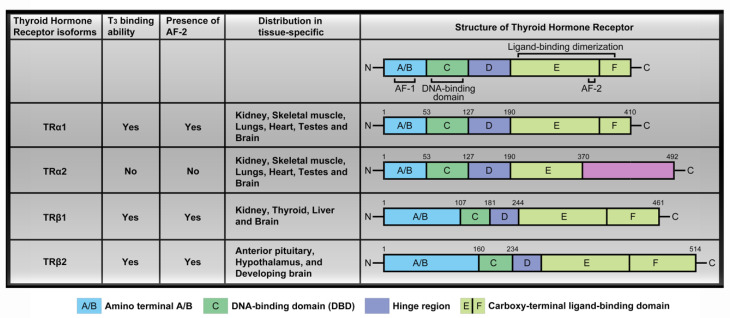
Thyroid hormone receptors (TR) isoforms and structure distribution. Thyroid hormone receptors (TR) contain several domains, specifically, amino-terminal A/B that may function as a gene enhancer, DNA-binding domain (DBD), hinge region containing the nuclear localization signal and carboxy-terminal ligand-binding domain that binds T_3_. The four major TR isoforms, TRα1, TRα2, TRβ1, and TRβ2 undergo TH binding and are widely distributed in a tissue-specific manner, such as TRα1 and TRα2 are expressed in the kidney, skeletal muscle, lungs, heart, and testes, with particularly high levels detected in the brain. TRβ1 expression is significant in the brain, thyroid, liver, and kidney while the TRβ2 isoform is specifically expressed in the anterior pituitary, hypothalamus, and developing brain.
